# Assessing Disparities in Video-Telehealth Use and eHealth Literacy Among Hospitalized Patients: Cross-sectional Observational Study

**DOI:** 10.2196/44501

**Published:** 2023-05-12

**Authors:** Jessica Cheng, Vineet M Arora, Nicole Kappel, Hanna Vollbrecht, David O Meltzer, Valerie Press

**Affiliations:** 1 Pritzker School of Medicine University of Chicago Chicago, IL United States; 2 General Internal Medicine Department of Medicine University of Chicago Chicago, IL United States; 3 Internal Medicine Brigham and Women's Hospital Boston, MA United States; 4 Hospital Medicine Department of Medicine University of Chicago Chicago, IL United States

**Keywords:** health literacy, eHealth literacy, telehealth, transitions of care, digital divide, telemedicine, technology use, video consultation, urban population, digital health literacy, patient care

## Abstract

**Background:**

Medicare coverage for audio-only telehealth is slated to end this year after the public health emergency concludes. When the time comes, many patients may be unable to make the transition from audio-only to video telehealth due to digital inexperience. This study explores the *second* digital divide within video telehealth use, which is primarily characterized by skills and capabilities rather than access, by measuring eHealth literacy (eHL) and video capabilities in hospitalized patients.

**Objective:**

The aim of this study is to evaluate video capabilities, eHealth literacy, and engagement with video telehealth among hospitalized patients.

**Methods:**

The study design is a cross-sectional observational study of adult inpatients at the University of Chicago Medical Center. We assessed self-reported rates of audio versus video telehealth usage as well as the participants’ self-reported willingness to use video telehealth for future health care visits. We used a multivariable binary logistic regression to determine the odds ratio for being unwilling to use video telehealth, adjusted for age, sex, race or ethnicity, educational level, eHL literacy scale (eHEALS), health literacy (brief health literacy screen), technology access, internet access, and video capability.

**Results:**

Of the 297 enrolled participants, median age was 58 years, most (n=185, 62%) identified as Black, half (n=149, 50%) were female, one-quarter (n=66, 22%) lacked home internet access, and one-third (n=102, 34%) had inadequate eHL.

**Conclusions:**

Patients with low eHL reported greater participation in audio-only telehealth over video telehealth, of which the former may lose its flexible pandemic reimbursement policy. This may widen the existing health disparities as older adults and patients with low eHL face challenges in accessing video telehealth services. Low eHL is associated with lack of web-based skills, lower rates of video telehealth usage, and lower willingness to use video technology. The study results raise the question of how to improve video capability among patients who, despite having access to smartphones and laptops, face challenges in using telehealth optimally.

## Introduction

Telehealth emerged as a ubiquitous communication tool for providers and patients during the COVID-19 pandemic [1-3]. The number of Medicare telehealth visits increased from 840,000 in 2019 to nearly 53 million in 2020 [3]. Among these telehealth visits, up to 70% could be attributed to audio-only services [3]. This was in part due to an expansion in Medicare coverage during the public health emergency, which allowed for audio-only telehealth to increase access to virtual-based medical care [4].

Coverage for audio-only telehealth is slated to end after the public health emergency concludes [5]. Given the uncertain future of reimbursement for audio-only telehealth visits, it is important to investigate the disparities between audio-only and video telehealth usage. Video-enabled visits present the advantage of additional visual assessments and increased engagement. However, they also require increased digital capability from the patient to go online and operate video technology [6,7]. Recent evidence has shown that there are significant disparities when comparing audio-only to video telehealth use. Video telehealth use is lowest in patients who are non-White and older and who have less educational attainment [2,8,9].

Understanding the mediating factors behind video telehealth use and the unwillingness to use video telehealth can help inform telehealth policy going forward. Given the near ubiquity of smartphone ownership across demographics, there is a potential for video telehealth to have a broad reach [10,11]. However, research has shown that patients face gaps in technology capability, despite increases in technology ownership [12-14]. A study estimated that more than one-third of older adults in 2018 were not ready for video telehealth visits, predominately due to digital inexperience [6].

The intersection of health literacy and digital literacy may be particularly relevant in understanding the *second* digital divide within video telehealth, which is primarily characterized by skills and capabilities rather than access [15]. eHealth literacy (eHL) is the digital literacy skill to seek out and use health information from electronic sources, extending the concept of health literacy into the digital realm [16]. Prior research at an urban site showed that the majority of patients with low health literacy had access to technology and internet, but low eHealth literacy [17]. To date, we have not identified prior exploration of the role of eHL in the disparity between audio-only and video telehealth use. This study examines video capability and telehealth usage in a hospitalized, urban population, stratified by eHL.

## Methods

### Study Design

We conducted a cross-sectional observational study among adult inpatients at the University of Chicago Medical Center, as part of the University of Chicago Hospitalist Project, a larger ongoing quality-of-care study [18]. We surveyed participants from August 2020 through March 2022.

Eligibility criteria included hospitalization on general medicine services, being 18 years or older, being English speaking, and consenting to the primary study.

### Ethical Considerations

The University of Chicago Biological Sciences Division Institutional Review Board approved this protocol (#IRB16-0763). The participants were consented for primary data collection and secondary analysis of research data. Study data are deidentified.

### Measures

The primary outcome variables were self-reported audio-only and video-enabled telehealth usage and willingness to use video telehealth, captured by binary survey responses. The participants were asked if they had a telehealth appointment prior to the pandemic (defined as before March 2020), if they had a telehealth appointment since the pandemic (defined as after March 2020), and whether they were willing to use video telehealth visits.

As mentioned, technology access and home internet access were self-reported and captured by binary survey responses. To assess technology capabilities, the participants were asked if they knew how to perform given web-based tasks, including the capability to use video without needing help.

The participants were categorized as having either low eHL or adequate eHL based on the eHealth literacy scale (eHEALS), a validated 8-item questionnaire with each item scored on a 5-point Likert scale [16]. The resultant composite score is between 8 and 40. Following other relevant studies, low eHL was defined as a score of <26, and adequate eHL was defined as a score of ≥26 [19,20].

Health literacy (HL) was based on the brief health literacy screen, which is a 3-item survey scored on a Likert scale from 0 to 4 [21]. Low HL is defined by scoring 2 or less on any item in the brief health literacy screen.

### Data Analysis

We assessed self-reported telehealth usage (audio-only versus video-enabled), willingness to use video visits and technology access, as well as demographic characteristics, across eHL levels using descriptive statistics. Chi-squared tests were used to calculate *P* values. Statistical significance was set at *P*<.05.

We then used a multivariable binary logistic regression to determine the odds ratio for being unwilling to use video telehealth, adjusted for age, sex, race or ethnicity, educational level, eHL, HL, technology access, internet access, and technology capability. All analyses were performed using STATA version 16.1 (StataCorp LLC).

## Results

### Participant Characteristics

Of the 297 adults included in this study, the majority identified as Black (n=185, 62%), half identified as female (n=149, 50%), and the median age was 58 years (25th percentile to 75th percentile: 42-68; Table 1). The median eHEALS score was 30 (25th percentile to 75th percentile: 21-33; Table 1).

Participants with adequate eHL had significantly higher rates of college educational attainment (79/196, 40% vs 18/101, 18%), smartphone ownership (186/196, 95% vs 64/101, 63%), laptop ownership (122/196, 62% vs 20/101, 20%), tablet ownership (79/196, 40% vs 20/101, 20%), and video capability (185/196, 91% vs 48/101, 48%) and fewer households without internet access (26/196, 13% vs 40/101, 40%) compared with participants with low eHL (all *P*<.001; Table 1).

**Figure 1:**
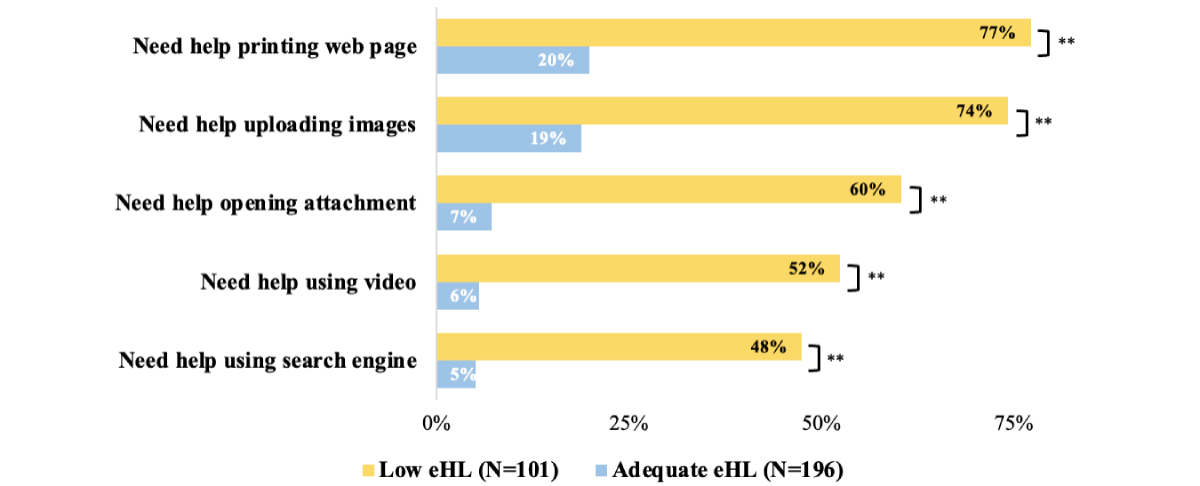
Percentage of participants who report needing help with web-based tasks by eHealth literacy (eHL) level. **P<.001.

Low eHL was associated with lower rates of web-based skills, such as the ability to use video without help (*P*<.001; Figure 1). Participants with low eHL were more likely to need help with using video technology than those with adequate eHL (53/101, 52% vs 11/196, 6%; *P*<.001; Figure 1).

**Table 1:** Participant demographics and technology access by eHealth literacy (eHL) level with bivariate *P* values.

Demographics			Values						
			All participants (n=297)		Adequate^a^ (n=196)		Low eHL^a^ (n=101)		*P* value
Age, median (Q25-Q75)			58 (42-68)		53 (38-66)		64 (52-74)		<.001
Age ≥65 years, n (%)			108 (36)		58 (30)		50 (50)		.001
Female, n (%)			149 (50)		104 (53)		45 (45)		.17
**Race, n (%)**									.005
	White	74 (25)		60 (31)		14 (14)			
	Black	185 (62)		111 (57)		74 (73)			
	Other^b^	38 (13)		25 (13)		13 (13)			
**Education, n (%)**									<.001
	College	97 (33)		79 (40)		18 (18)			
	High school	166 (56)		106 (54)		60 (59)			
	<High school	31 (10)		10 (5)		21 (21)			
	Refused	3 (1)		1 (1)		2 (2)			
eHL, mean (Q25-Q75^c^)			27 (21-33)		33 (30-35)		15 (8-21)		<.001
Low HL^d^, n (%)			102 (34)		50 (26)		52 (51)		<.001
**Technology access, n (%)**									<.001
	Own smartphone	250 (84)		186 (95)		64 (63)			
	Own laptop	142 (48)		122 (62)		20 (20)			
	Own tablet	99 (33)		79 (40)		20 (20)			
	No home internet access, n (%)	66 (22)		26 (13)		40 (40)			
	Need help using video, n (%)	64 (22)		11 (6)		53 (52)			

^a^eHEALS is an 8-item survey with a composite score of 40. Low eHL is defined by a score of <26, and adequate eHL is defined by a score of ≥24. Significance level is set as *P*<.05.

^b^The category of other included participants who self-reported their race as American Indian, Alaskan Native, Asian, multiple ethnicities, don’t know, or refused.

^c^Q25: 25th percentile; Q75: 75th percentile.

^d^HL: health literacy; HL is based on the 3-item brief health literacy screen (BHLS), which is scored on a Likert scale from 0 to 4. Low HL is defined by scoring 2 or less on any item in the BHLS.

### Video Telehealth Appointments During COVID-19

**Figure 2:**
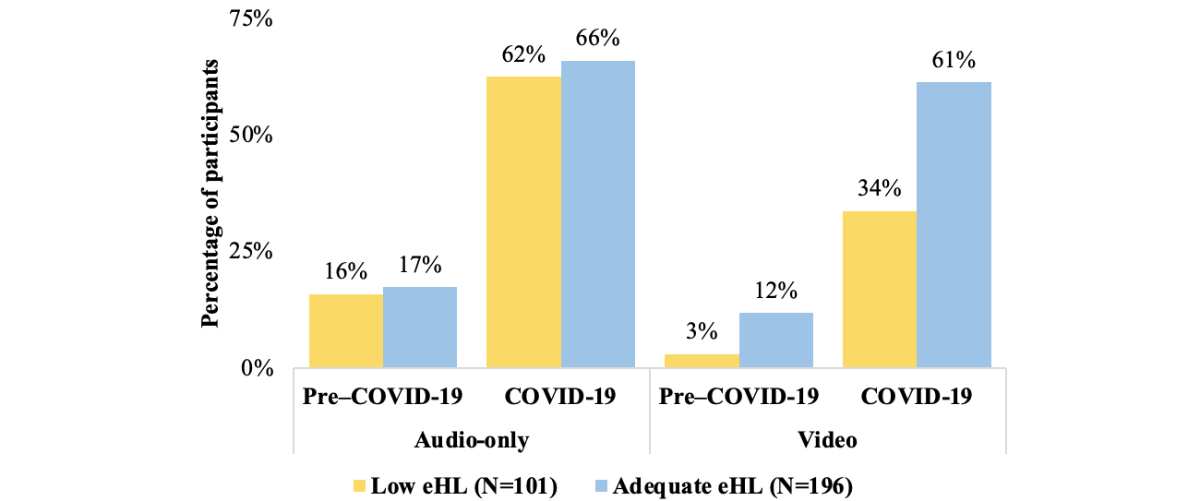
Percentage of participants reporting telehealth appointments via audio-only or video technology before March 2020 (pre–COVID-19) and as of March 2020 (COVID-19). eHL: eHealth literacy; HL: health literacy. *P<.05.

Fewer participants with low eHL reported a video telehealth appointment compared with those with adequate eHL (34/101, 34% vs 120/196, 61%; *P*=.007; Figure 2). In contrast to video visits, audio-only telehealth usage during the pandemic did not differ significantly between eHL subpopulations (63/101, 62% vs 129/196, 66%; *P*=.56).

### Predictors for Unwillingness to Use Video Telehealth

Using a multivariable binary logistic regression, we found that unwillingness to use video technology for telehealth visits was significantly associated with older age (*P*=.03), low eHL (*P*=.003), lack of video capability (ie, needing help using video; *P*=.04), and lack of home internet access (*P*=.03; Table 2). In contrast to eHL, HL was not a significant predictor for unwillingness to use video technology (*P*=.48; Table 2).

**Table 2:** Adjusted odds of telehealth unwillingness for video visits by demographic and technology factors.

Factor		Adjusted odds ratio (95% CI)	*P* value	
**Age (years)**				
	<65	1 (reference)		
	≥65	1.9 (1.1-3.5)	.03	
**Sex**				
	Male	1 (reference)		
	Female	1.3 (0.7-2.2)	.43	
**Race**				
	White	1 (reference)		
	Black	0.9 (0.4-1.8)	.72	
	Other	1.5 (0.6-3.8)	.38	
**Educational level**				
	College	1 (reference)		
	High school	1.3 (0.7-2.4)	.45	
	>High school	2.0 (0.7-5.6)	.18	
Low eHL^a^		2.7 (1.4-5.3)	.003	
Low HL^b^		0.8 (0.4-1.5)	.48	
Need help using video		2.5 (1.1-5.9)	.04	
**Technology access**				
	Own smartphone	1.1 (0.4-2.8)	.81	
	Own laptop	0.9 (0.5-1.7)	.70	
	Own tablet	0.8 (0.4-1.5)	.47	
	No home internet access	2.3 (1.1-4.8)	.03	

^a^eHL: eHealth literacy.

^b^HL: health literacy. Significance level was set at *P*<.05.

## Discussion

### Principal Findings

Telehealth adoption increased across the board during the pandemic. However, our study found significant disparities in telehealth usage when comparing the 2 telehealth modalities. Patients with low eHL reported greater participation in audio-only telehealth over video telehealth, of which the former may lose its flexible pandemic reimbursement policy. This has the potential to widen the existing health disparities as older adults and patients with low eHL face challenges in transitioning to video telehealth services. The use of audio-only visits was similar across most demographic subgroups; however, the rates of video visits and self-reported ability to use video technology varied significantly by eHL level.

Our findings are consistent with prior studies showing disparities in audio-only versus video telehealth use during the pandemic [2,8,9]. Video telehealth requires additional access to computers and home broadband or smartphones. However, Black and Hispanic adults and adults from low-income households are less likely to report access to home broadband and computers [10,22,23]. Some reasons for low use of video visits are rooted in historical redlining policies that denied services to low-income communities and communities of color, and, more recently, the “digital redlining” of broadband as internet service providers build new fiber networks [24,25]. The ramifications of this on equitable digital patient care is of particular importance in an increasingly digital environment.

Beyond the gaps in broadband access and technology ownership, importantly, we show that gaps in digital skills, specifically low electronic health literacy, were also a driver in video telehealth disparities. Low eHL and lack of video capability reduced the likelihood of being willing to engage in video-enabled telehealth. After multivariable adjustment, the willingness to use video telehealth was significantly lower among participants who were older, lacked home internet access, had low eHL, and needed help using video technology. These results are consistent with prior literature that demonstrate how patients still face challenges in knowing how to use video technology to improve their health despite having access to smartphones and internet [14,17,26].

### Limitations and Strengths

There are several limitations to this study. This was an urban, single-site study; therefore, the results may not be generalizable to rural locations where a greater proportion of patients may lack broadband access. The study includes only English-speaking patients, so the findings may differ among patients with limited English proficiency who may face additional challenges in telehealth use. Additionally, the study uses patient self-reported measures for telehealth use and technology ownership. While this could introduce recall and social desirability bias, our study design has several strengths. The self-reported nature of the study allows for additional information on patient telehealth preferences that is not captured in claims data. Moreover, the survey was administered over the phone, which reduces the chance of bias from internet surveys that self-select for patients with greater technology capability.

Our study contributes to the literature by showing the positive association of adequate eHL with the patients’ willingness to engage in video visits, and conversely, the negative relationship between low eHL and video engagement. The study results raise the question of how to improve video capabilities and skills among patients who have access to smart devices but face challenges in accessing the internet and operating video equipment. Moving forward, additional research should investigate what interventions will increase digital skills, particularly eHealth literacy, in patients who encounter barriers to using video telehealth.

## Abbreviations

eHEALS: eHealth literacy scale
eHL: eHealth literacy
HL: health literacy

## Data Availability

Since the data used in this manuscript are highly granular and contain potentially sensitive patient information, the public sharing of the data would breach the University of Chicago’s institutional review board protocol requirements. Interested researchers may contact author VP via email for data requests.

## References

[1] Park J, Erikson C, Han X, Iyer P (2018). Are State Telehealth Policies Associated With The Use Of Telehealth Services Among Underserved Populations?. Health Aff (Millwood).

[2] Karimi M, Lee E, Couture S, Gonzales A, Grigorescu V, Smith SR, De Lew N, Sommers BD National Survey Trends in Telehealth Use in 2021: Disparities in Utilization and Audio vs Video Services. Assistant Secretary for Planning and Evaluation.

[3] Samson L, Tarazi W, Turrini G, Sheingold S Medicare Beneficiaries? Use of Telehealth in 2020: Trends by Beneficiary Characteristics and Location. Assistant Secretary for Planning and Evaluation.

[4] Medicare telemedicine health care provider fact sheet. CMS.gov.

[5] CY2022 Telehealth Update Medicare Physician Fee Schedule. Medical Learning Network.

[6] Lam K, Lu AD, Shi Y, Covinsky KE (2020). Assessing Telemedicine Unreadiness Among Older Adults in the United States During the COVID-19 Pandemic. JAMA Intern Med.

[7] Chang JE, Lindenfeld Z, Albert SL, Massar R, Shelley D, Kwok L, Fennelly K, Berry CA (2021). Telephone vs. Video Visits During COVID-19: Safety-Net Provider Perspectives. J Am Board Fam Med.

[8] Chen J, Li KY, Andino J, Hill CE, Ng S, Steppe E, Ellimoottil C (2022). Predictors of Audio-Only Versus Video Telehealth Visits During the COVID-19 Pandemic. J Gen Intern Med.

[9] Rodriguez JA, Betancourt JR, Sequist TD, Ganguli I (2021). Differences in the use of telephone and video telemedicine visits during the COVID-19 pandemic. Am J Manag Care.

[10] Atske S, Perrin A Home broadband adoption, computer ownership vary by race, ethnicity in the U.S. Pew Research Center.

[11] Demographics of Mobile Device Ownership and Adoption in the United States. Pew Research Center.

[12] Mackert M, Mabry-Flynn A, Champlin S, Donovan EE, Pounders K (2016). Health Literacy and Health Information Technology Adoption: The Potential for a New Digital Divide. J Med Internet Res.

[13] Sarkar U, Karter AJ, Liu JY, Adler NE, Nguyen R, Lopez A, Schillinger D (2010). The literacy divide: health literacy and the use of an internet-based patient portal in an integrated health system-results from the diabetes study of northern California (DISTANCE). J Health Commun.

[14] Chesser A, Burke A, Reyes J, Rohrberg T (2016). Navigating the digital divide: A systematic review of eHealth literacy in underserved populations in the United States. Inform Health Soc Care.

[15] van Deursen A, van Dijk J (2010). Internet skills and the digital divide. New Media & Society.

[16] Norman CD, Skinner HA (2006). eHEALS: The eHealth Literacy Scale. J Med Internet Res.

[17] Vollbrecht H, Arora V, Otero S, Carey K, Meltzer D, Press VG (2020). Evaluating the Need to Address Digital Literacy Among Hospitalized Patients: Cross-Sectional Observational Study. J Med Internet Res.

[18] Meltzer D, Manning WG, Morrison J, Shah MN, Jin L, Guth T, Levinson W (2002). Effects of physician experience on costs and outcomes on an academic general medicine service: results of a trial of hospitalists. Ann Intern Med.

[19] Richtering SS, Hyun K, Neubeck L, Coorey G, Chalmers J, Usherwood T, Peiris D, Chow CK, Redfern J (2017). eHealth Literacy: Predictors in a Population With Moderate-to-High Cardiovascular Risk. JMIR Hum Factors.

[20] Shiferaw KB, Mehari EA (2019). Internet use and eHealth literacy among health-care professionals in a resource limited setting: a cross-sectional survey. Adv Med Educ Pract.

[21] Chew LD, Griffin JM, Partin MR, Noorbaloochi S, Grill JP, Snyder A, Bradley KA, Nugent SM, Baines AD, Vanryn M (2008). Validation of screening questions for limited health literacy in a large VA outpatient population. J Gen Intern Med.

[22] Press VG, Huisingh-Scheetz M, Arora VM (2021). Inequities in Technology Contribute to Disparities in COVID-19 Vaccine Distribution. JAMA Health Forum.

[23] Digital divide persists even as Americans with lower incomes make gains in tech adoption. Pew Research Center.

[24] Tibken S The broadband gap?s dirty secret: Redlining still exists in digital form. CNET.

[25] Falcon E The FCC and States Must Ban Digital Redlining. Electron Frontier Foundation.

[26] Vollbrecht H, Arora VM, Otero S, Carey KA, Meltzer DO, Press VG (2021). Measuring eHealth Literacy in Urban Hospitalized Patients: Implications for the Post-COVID World. J Gen Intern Med.

